# Cerebral glucose hypometabolism and hypoperfusion of cingulate gyrus: an imaging biomarker of autoimmune encephalitis with psychiatric symptoms

**DOI:** 10.1007/s00415-023-12051-z

**Published:** 2023-11-09

**Authors:** Yueqian Sun, Gongfei Li, Xiao Liu, Xiaobin Zhao, Jiechuan Ren, Guoping Ren, Yaou Liu, Lin Ai, Qun Wang

**Affiliations:** 1 Department of Neurology, Beijing Tiantan Hospital, Capital Medical University, No. 119 South 4th Ring West Road, Fengtai District, Beijing, 100070 China; 2 Department of Neurology & Stroke, University of Tübingen, Tübingen, Germany; 3 Department of Nuclear Medicine, Beijing Tiantan Hospital, Capital Medical University, Beijing, China; 4 Department of Radiology, Beijing Tiantan Hospital, Capital Medical University, Beijing, China; 5 National Center for Clinical Medicine of Neurological Diseases, Beijing, China; 6 Beijing Institute of Brain Disorders, Collaborative Innovation Center for Brain Disorders, Capital Medical University, Beijing, China

**Keywords:** Autoimmune encephalitis, Psychiatric symptoms, Cingulate, Positron emission tomography, Arterial spin labeling

## Abstract

**Background:**

About 60% of autoimmune encephalitis (AE) patients present psychiatric symptoms, but the underlying mechanism remains unknown. This study examined the role of the cingulate cortex in such patients to identify predictive poor psychiatric factors.

**Methods:**

In this study, 49 AE patients and 39 healthy controls were enrolled. AE patients were further divided into two groups based on the presence/absence of psychiatric symptoms. The ratio of the standardized uptake value (SUVR) and relative cerebral blood flow (rCBF) in different regions of the cingulate cortex were calculated through positron emission tomography–computed tomography (PET/CT) and arterial spin labeling (ASL) MRI, and the results were compared among the three groups. In addition, we followed-up on the psychiatric outcomes and identified the risk factors for poor psychiatric prognosis, focusing on the cingulate cortex.

**Results:**

More than half of the AE patients (27/49) exhibited psychiatric symptoms. Agitation and thought blocking were typical psychiatric phenotypes, except for anti-glutamic acid decarboxylase 65 (GAD65) encephalitis, which mainly presented with catatonia and a depressed mood. AE patients with psychiatric symptoms experienced reduced metabolism and perfusion of the anterior cingulate cortex (ACC), midcingulate cortex (MCC), and posterior cingulate cortex (PCC). The SUVR of ACC can be used as an independent risk factor of poor psychiatric outcomes, which had an area under the ROC curve (AUC) of 0.865.

**Conclusion:**

Impaired cingulate cortex function in AE may be the potential mechanism of psychiatric symptoms. Hypometabolism of ACC is an independent prognostic factor predicting an unfavorable psychiatric prognosis in AE.

**Supplementary Information:**

The online version contains supplementary material available at 10.1007/s00415-023-12051-z.

## Introduction

Autoimmune encephalitis (AE), a heterogeneous group of neurological autoimmune disorders, is associated with cognitive and psychiatric symptoms [1]. A cohort study of 100 AE people reported that 60% of individuals mainly presented with psychiatric symptoms, which remained significantly predominant throughout the study period [2]. About a third of these patients required inpatient services for their altered psychiatric status as the initial manifestation. Psychiatric symptoms are mainly characterized by affective and psychotic manifestations, including hallucinations, delusions, agitation, thought blocking, catatonia, and a depressed mood. Many of these patients were initially assessed by psychiatrists or admitted to psychiatric centers. A more detailed understanding of the phenomenology of psychiatric presentations is required to encourage clinicians to consider AE as a potential diagnosis. Although the distinct characteristics of psychiatric symptoms in AE are easily recognizable, the underlying causes remain unknown.

After recovery, most acute AE patients experience permanent psychiatric effects, such as depression and personality changes. Psychiatric symptoms can negatively impact the patient’s cognitive function. However, clinical predictors of psychiatric outcomes in AE remain unclear. A quantifiable and specific neuroimaging biomarker may help uncover the pathogenesis of AE and provide a potential prognostic evaluation of psychiatric symptoms in such patients. The cingulate gyrus is believed to play a central role in psychiatric illness [3, 4]. Previous studies have examined the impact of the cingulate cortex on psychiatric illnesses such as schizophrenia [5, 6], depression [7], anxiety disorders [8], catatonia [9], obsessive–compulsive disorder [10], and so on. Based on prior research, in this study, we selected the cingulate cortex as the region of interest (ROI) to understand the psychiatric symptoms in AE.

Two functional imaging techniques, ^18^F-fluorodeoxyglucose positron emission tomography–computed tomography scan (^18^F-FDG-PET/CT) and arterial spin labeling (ASL) MRI, have been used by previous studies [11–15] to observe perfusion and metabolism changes in AE. ^18^F-FDG-PET is a multimodal molecular imaging technique that combines functional metabolic and anatomical images, while ASL quantifies cerebral blood flow and perfusion alterations without using exogenous contrast agents. These two techniques, ^18^F-FDG-PET and ASL MRI, are closely linked as they can examine the two strongly coupled aspects of the central nervous system: perfusion and metabolism [16]. An integrated approach with MRI perfusion and PET metabolism may assist clinicians in planning personalized management strategies. Therefore, here, we used a combination of ASL MRI and PET to validate the role of the cingulate cortex in AE patients with psychiatric symptoms.

We performed perfusion/metabolism analysis to: (i) examine the characteristics of psychiatric symptoms in AE patients with different antibody types; (ii) investigate the role of the cingulate cortex in AE patients with psychiatric symptoms; and (iii) identify the independent predictive factors for poor psychiatry outcomes in AE.

## Materials and methods

### Participants

**Fig. 1:**
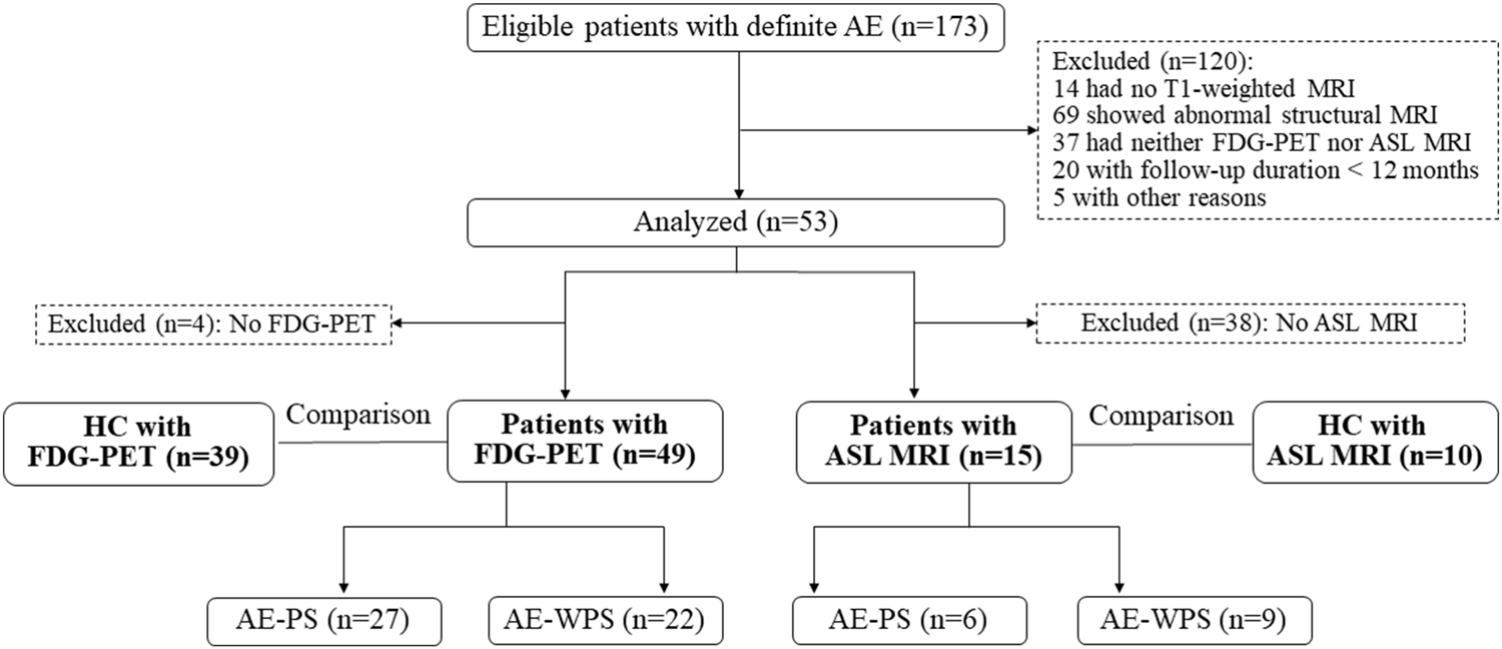
Flowchart of participants selection. *FDG-PET* fluorodeoxyglucose positron emission tomography, *ASL* arterial spin labeling, *HC* healthy controls, *AE* autoimmune encephalitis, *AE-PS* autoimmune encephalitis with psychiatric symptoms, *AE-WPS* autoimmune encephalitis without psychiatric symptoms

We retrospectively reviewed patients diagnosed with AE in Beijing Tiantan Hospital between Match 2019 and Match 2022 (Fig. 1). The inclusion criteria were as follows: Age, 18–80 years old; positive antibodies in serum and/or cerebrospinal fluid (CSF) [1]; ^18^F-FDG-PET or ASL MRI scans; and follow-up duration > 12 months (unless the patient died from AE). The exclusion criteria were as follows: structural abnormalities in conventional MRI; seizures or subclinical seizures within 2 h before and after the PET examination; FDG-PET images with insufficient glucose uptake; FDG-PET images and ASL MRI with motion artifacts; and any missing data. We excluded patients with MRI lesions to avoid the impact of structural abnormalities on brain metabolism. Furthermore, in daily clinical practice, AE is often misdiagnosed as primary psychosis when MRI results are negative [17]. All patients were followed-up after at least 1 year from symptom onset and classified into two groups based on the absence or presence of psychiatric symptoms (patients with a good psychiatric prognosis and patients with a poor psychiatric prognosis).

### Healthy controls

A group of 39 healthy, age- and sex-matched participants were enrolled from the FDG-PET database of Beijing Tiantan Hospital. Ten of them were selected as healthy controls for ASL MRI analysis. The healthy control group had no history of neuropsychiatric disease, including, but not limited to, underlying brain injury, stroke, brain tumors, dementia, Parkinsonism, ataxia, epilepsy, and multiple sclerosis.

### ^18^F-FDG-PET and ASL MRI

PET images were acquired using a PET/CT scanner (Elite Discovery, GE HealthCare, USA) with a matrix size of 192 × 192 and a slice thickness of 3.27 mm. Patients fasted for at least 6 h and their serum glucose levels were maintained below 8 mmol/L. Patients were administered an intravenous injection of 310 MBq/70 kg of body weight of 18F-FDG. PET data reconstruction was performed using the ordered subset expectation maximization (OSEM) algorithm.

ASL scans were acquired using the 3D pseudo-continuous arterial spin labeling technique on a 3 T Siemens Magnetom Prisma MRI scanner (Siemens Healthineers, Germany) with a 64-channel head coil. No contrast agents were used, and patients were required to be stable during scanning. The scanning parameters were as follows: repetition time (TR) = 4705 ms; echo time (TE) = 11.2 ms; field of view (FOV) = 128 × 128 mm; number of excitations (NEX) = 1; 30 × 4.0 mm axial sections with whole brain coverage; scan duration, 6 min and 14 s.

### Image analysis

After the acquisition of ^18^F-FDG-PET and ASL images, all data were processed using MATLAB 2019b software with statistical parametric mapping 12 (SPM12) software. The cingulate gyrus, including the anterior cingulate cortex (ACC), the midcingulate cortex (MCC), and the posterior cingulate cortex (PCC), was selected as ROI based on the Montreal Neurological Institute (MNI) template, which was subsequently transformed into masks.

PET data preprocessing steps were as follows: first, the PET images were spatially normalized into MNI atlas anatomical space following a 12-parameter affine transformation and nonlinear transformations. This yielded images of 2 mm × 2 mm × 2 mm voxels. Next, to enhance the signal-to-noise ratio, the default SPM smoothing was applied using an 8-mm Gaussian kernel. Finally, the ratio of the standardized uptake value (SUVR) was calculated from the voxel size-weighted median FDG uptake within selected ROIs, normalized to the cerebellum.

After conducting preliminary analyses on FDG-PET data, we focused on ASL data for subsequent analyses. The relative cerebral blood flow (rCBF) values of all selected ROIs were calculated from the CBF map of ASL, which represents the ratio of ROI to the cerebellum. To identify the metabolism and perfusion changes in the cingulate cortex, statistical analyses were performed with a one-way analysis of variance (one-way ANOVA) to compare patients and the control group, followed by Tukey’s multiple comparisons test. SUVR data were compared between groups of different psychiatric prognoses using a *t* test, while the rCBF was not analyzed because of the small sample size.

### Statistical analysis

In this study, we employed several methods for data analysis. Continuous variables are reported as the median (interquartile range; IQR). Categorical variables are presented as frequencies with corresponding percentages. We compared groups (patients with psychiatric symptoms and patients without psychiatric symptoms) using a *t* test for continuous variables that were normally distributed (calculated with the Shapiro–Wilk test), Mann–Whitney *U* tests for non-parametric data, and *c*^2^ tests or Fisher’s exact tests for categorical variables. The differences in metabolism and perfusion among the three groups were analyzed using one-way ANOVA and the Tukey test for multiple comparisons. Clinical variables were comprehensively collected for possible inclusion in the risk model of psychiatric prognosis. We used binary logistic regression to assess independent risk factors associated with AE with a long-term poor psychiatric prognosis. Then all variables with *p* < 0.2 in the univariate analysis were included in the multivariable logistic regression model. Subsequently, the likelihood ratio test was used in a backward elimination process (*p* < 0.05 to retain, *p* > 0.1 to remove) to select the final set of independent risk factors for inclusion in the model. The odds ratio (OR) with a 95% confidence interval (CI) was presented for the logistic regression model. A two-sided *p* < 0.05 was considered to indicate statistical significance. SPSS 22.0 software package (IBM Corp., Armonk, New York, USA) and Prism 8 (GraphPad Software, CA, USA) were used for statistical analyses.

## Results

### Comparison of clinical characteristics between the two AE groups with different psychiatric status

A total of 49 patients (32 males and 17 females) with AE were enrolled in this study. Among these, 27 patients presented psychiatric symptoms (referred to as the AE-PS group), while 22 patients (referred to as the AE-WPS) were without them. There were no significant differences in terms of age and gender between the two groups; the median age ranged from 50 to 60 years, with a male predominance (Table 1). Almost all patients developed seizures and memory deficits, regardless of whether they were suffering from psychiatric symptoms (AE-PS, *p* = 0.196; AE-WPS, *p* = 0.282). Importantly, AE-PS patients were more likely to have sleep disorders (AE-PS, 48.15%; AE-WPS, 18.18%, *p* = 0.038) and a smoking history (AE-PS, 33.33%; AE-WPS, 4.55%, *p* = 0.015). No significant differences were found in any laboratory test of CSF or serum between the two AE groups. Besides, the relapse rate and functional prognosis showed no association with the presence of psychiatric symptoms (AE-PS, *p* = 0.590; AE-WPS, *p* = 0.534).

**Table 1:** Clinical characteristics of groups with different psychiatric status

	With psychiatric symptoms (*n* = 27)	Without psychiatric symptoms (*n* = 22)	*p*
Age, y	57 (45–67)	53 (30–62)	0.273
Male, *n* (%)	19 (70.37)	13 (59.09)	0.548
Smoking, *n* (%)	9 (33.33)	1 (4.55)	0.015^*^
Drinking, *n* (%)	8 (29.63)	2 (9.09)	0.152
FBDS, *n* (%)	11 (40.74)	4 (18.18)	0.123
Seizure, *n* (%)	27 (100.00)	20 (90.90)	0.196
Cognitive impairment, *n* (%)	14 (82.35) (10 missed)	13 (61.90) (1 missed)	0.282
Sleep disorders, *n* (%)	13 (48.15)	4 (18.18)	0.038^*^
CSF oligoclonal band, *n* (%)	14 (51.85)	12 (54.55)	> 0.999
CSF WBC count, cells/uL	6.50 (4.00–19.75)	4.00 (2.00–14.50)	0.068
CSF protein, mg/dL	45.75 (30.43–54.85)	38.54 (26.17–58.95)	0.461
CSF antibody titer	32 (10–32)	10 (1–100)	0.607
Serum antibody titer	32 (10–100)	32 (10–100)	0.273
Antibody subtype			
NMDAR	5 (18.52)	5 (22.73)	0.737
LGI1	16 (59.26)	10 (45.45)	0.397
GAD65	1 (3.70)	6 (27.27)	0.035^*^
GABAB	5 (18.52)	1 (4.55)	0.204
Abnormal EEG, *n* (%)	21 (77.78)	11 (50.00)	0.070
Tumor, *n* (%)	3 (11.11)	3 (13.64)	> 0.999
PET scan before treatment	13 (48.15)	9 (40.91)	0.774
Follow-up duration, m	32 (27 to 37)	28 (19 to 47)	0.932
mRS (follow-up)	1 (0 to 2) (4 missed)	1 (0 to 1) (2 missed)	0.534
Relapse, *n* (%)	1 (4.35) (4 missed)	2 (10.00) (2 missed)	0.590

Categorical variables were recorded as *n* (%) and analyzed by Mann–Whitney *U* test. Continuous variables were recorded as median (IQR) and analyzed by *c*^2^ test (*n* > 5) or Fisher’s exact test (n ≤ 5)

*CSF* cerebrospinal fluid, *EEG* electroencephalography, *FBDS* faciobrachial dystonic seizure, *mRS* modified Rankin scale, *NMDAR* *N*-methyl-d-aspartate receptor, *LGI1* leucine-rich glioma-inactivated 1, *GAD65* glutamic acid decarboxylase 65, *GABAB* gamma-aminobutyric acid type B

### Psychiatric manifestations of AE with different antibodies

**Fig. 2:**
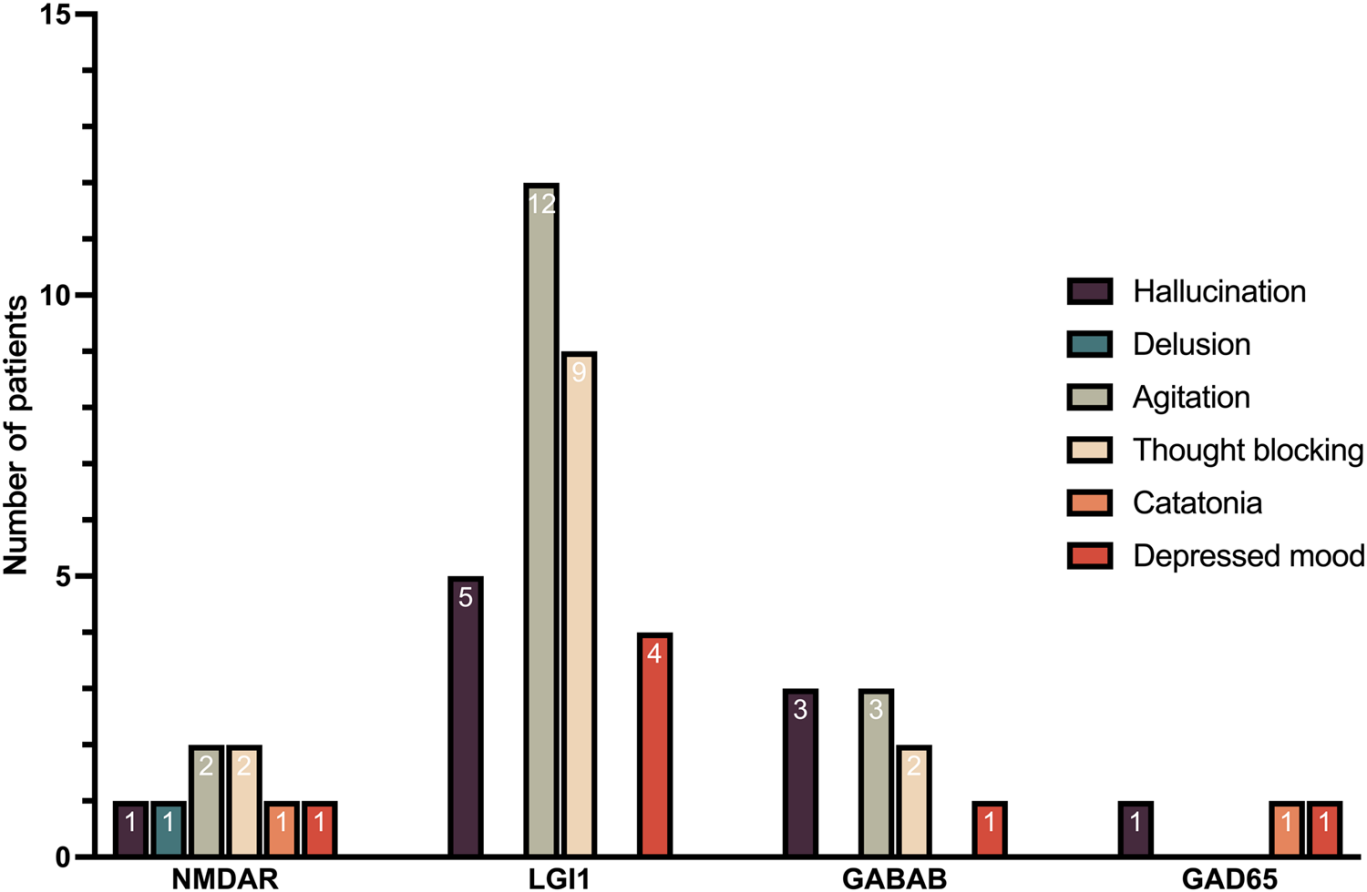
The psychiatric manifestations in autoimmune encephalitis with different antibodies. *NMDAR* *N*-methyl-d-aspartate receptor, *LGI1* leucine-rich glioma-inactivated 1, *GAD65* glutamic acid decarboxylase 65, *GABAB* gamma-aminobutyric acid type B

The highest prevalence of psychiatric symptoms was in patients with anti-gamma-aminobutyric acid type B (GABAB) encephalitis (83%), and the lowest was in patients with anti-glutamic acid decarboxylase 65 (GAD65) encephalitis (14%). Furthermore, about half of the patients with anti-*N*-methyl-d-aspartate receptor (NMDAR) encephalitis (50%) and anti-leucine-rich glioma-inactivated 1 (LGI1) encephalitis (62%) had psychiatric manifestations. Agitation and thought blocking were typical psychiatric phenotypes in all encephalitis with different antibodies (50% in anti-NMDAR encephalitis, 70% in anti-LGI1 encephalitis, and 56% in anti-GABAB encephalitis). However, in anti-GAD65 encephalitis, the predominant symptoms were catatonia and a depressed mood. In addition, hallucinations and delusions were less common in anti-NMDAR encephalitis and anti-LGI1 encephalitis compared with anti-GABAB encephalitis and anti-GAD65 encephalitis (Fig. 2).

### Cingulate metabolism and perfusion based on PET and MRI ASL

**Fig. 3:**
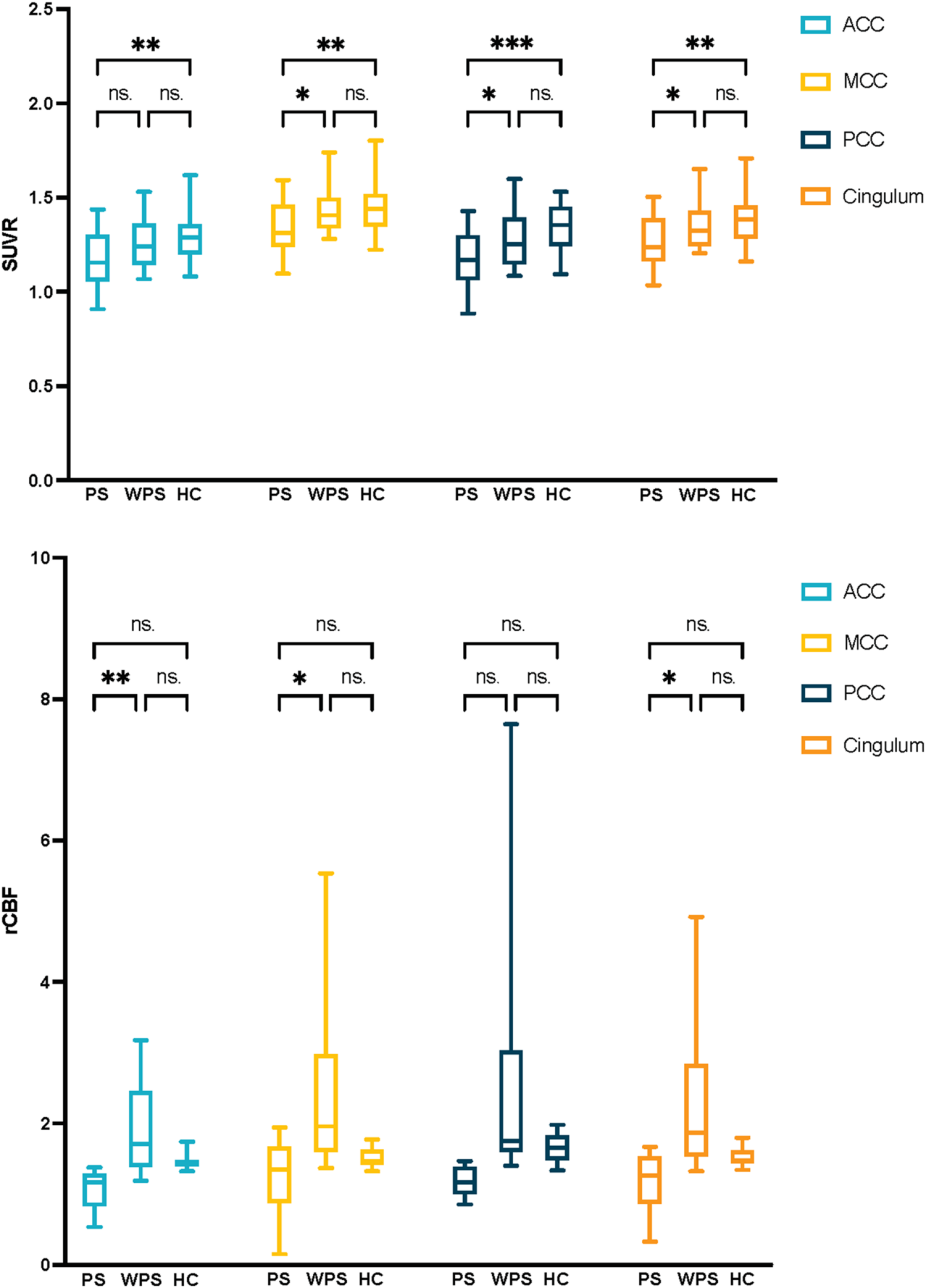
The comparison of SUVR and rCBF among three groups in different regions (ACC, MCC, PCC, and the whole cingulum). *PS* patients with psychiatric symptoms, *WPS* patients without psychiatric symptoms, *HC* healthy controls, *SUVR* the ratio of the standardized uptake value, *rCBF* relative cerebral blood flow, *ACC* anterior cingulate cortex, *MCC* midcingulate cortex, *PCC* posterior cingulate cortex. *p < 0.05, **p < 0.01, ***p < 0.001

In addition to 49 AE patients, we also included 39 healthy controls (HCs). Among them all, 39 underwent PET/CT scans and 10 underwent MRI ASL scans. Metabolic and perfusion differences among the three groups, AE-PS, AE-WPS, and HCs, were compared. Regarding SUVR (Fig. 3), AE-PS patients showed hypometabolism within the whole cingulate cortex, MCC, and PCC compared to AE-WPS patients and HCs. However, in comparison with HCs, ACC hypometabolism was found only in AE-PS patients. Regarding rCBF, AE-PS patients showed hypoperfusion within all parts of the cingulate cortex compared to AE-WPS patients, while there was no perfusion difference compared to HCs. Consistently, no difference in metabolism or perfusion was found between AE-WPS patients and HCs in any cingulum region.

### Psychiatric prognosis of AE and performance of the predictive model

**Fig. 4:**
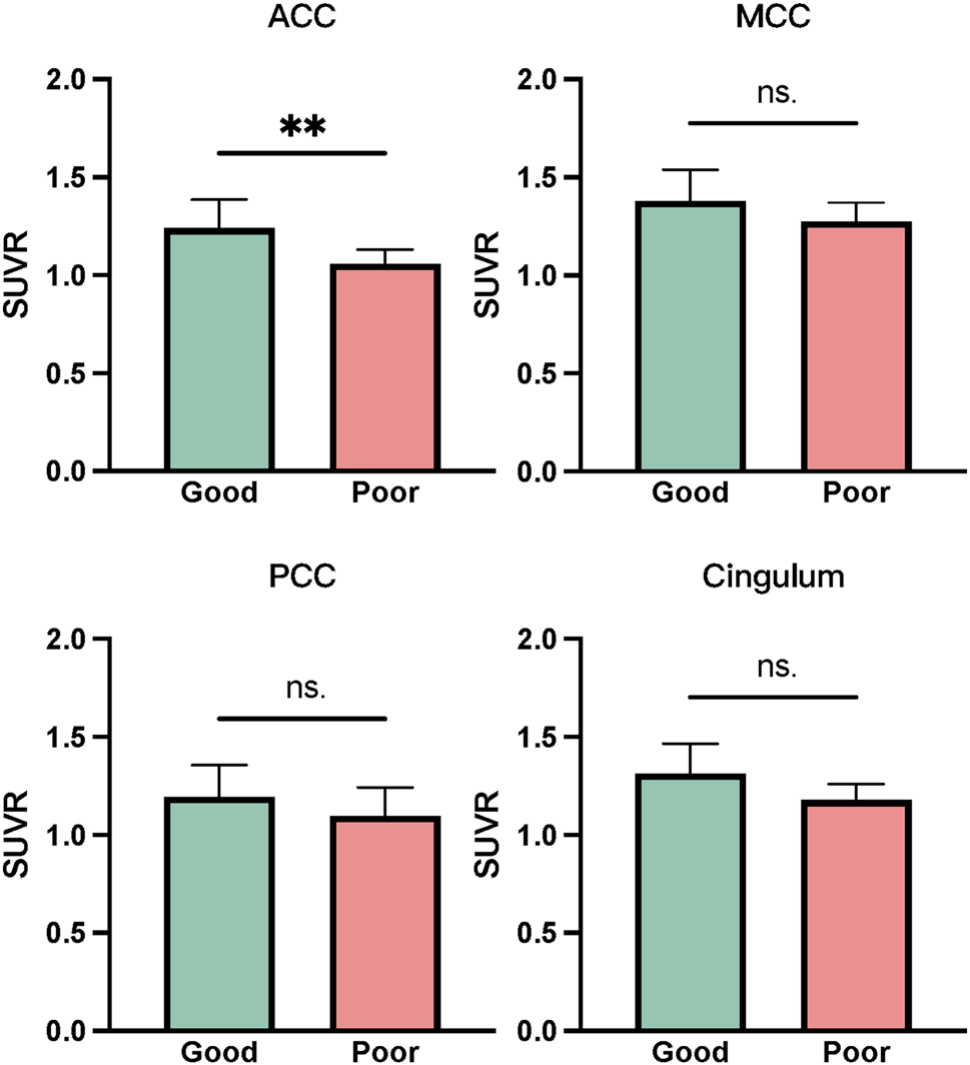
The comparison of SUVR between groups of different psychiatric prognosis. Good: good prognosis; Poor, poor prognosis. *SUVR* the ratio of the standardized uptake value, *ACC* anterior cingulate cortex, *MCC* midcingulate cortex, *PCC* posterior cingulate cortex. **p < 0.01

**Fig. 5:**
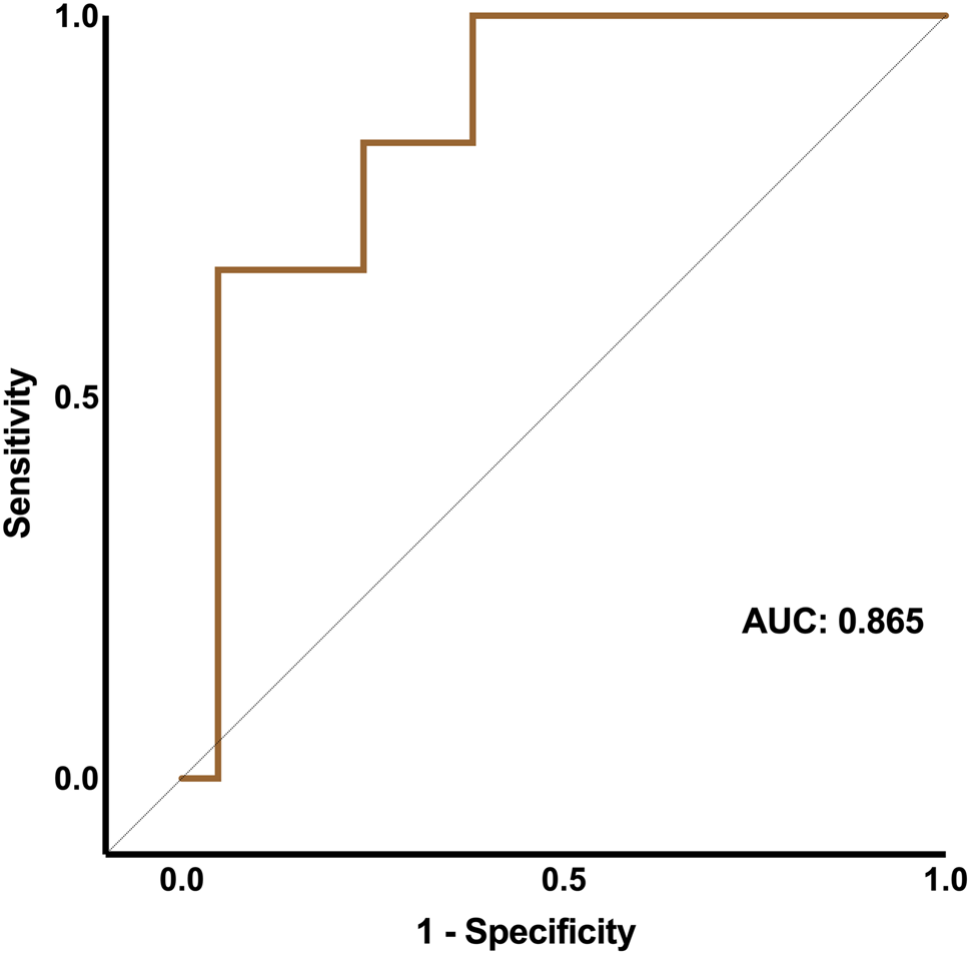
The ROC curve of multivariable regression model to predict poor psychiatric prognosis, which involved SUVR of ACC as the only predictor. ROC receiver operating characteristic, *AUC* area under the curve

Among the 27 AE-PS patients, 6 (22%) had a poor psychiatric prognosis, indicating that they were still suffering from mental-emotional abnormalities at follow-up. Three of them received anti-psychotic drugs, but the treatment failed, although they all had a good psychiatric prognosis after receiving immunotherapy. Treatment failure is defined as a lack of improvement in psychosis after adequate anti-psychotic drug treatment, including psychiatric rehospitalization, suicide attempt, discontinuation, switch to another medication, or death. As for these three patients, they all switched to immunotherapy [18]. Based on the SUVR comparison between the two groups and the results of univariate analysis, only glucose metabolism in ACC could independently predict poor psychiatric prognosis (*p* = 0.043) (Fig. 4). No significant correlation was detected between poor psychiatric prognosis and any other characteristics (Table 2). Subsequently, three predictors with *p* < 0.2, including CSF protein, CSF antibody titer, and SUVR of ACC, were selected to construct a multivariable logistic regression model. A receiver operating characteristic (ROC) curve was obtained, with an area under the curve (AUC) of 0.865, specificity of 0.952, sensitivity of 0.500, and accuracy of 0.852 (Fig. 5).

**Table 2:** Univariable and multivariable regression analysis of correlation between risk factors and poor psychiatric prognosis in autoimmune encephalitis

	Good psychiatric prognosis (*n* = 21)	Poor psychiatric prognosis (*n* = 6)	*p* (univariable)	*p* (multivariable)	OR (95% CI)
Age, y	52 (41–67)	58 (45–71)	0.519	–	–
Male, *n* (%)	15 (71.43)	4 (66.67)	0.822	–	–
Smoking, *n* (%)	8 (38.10)	2 (33.33)	0.832	–	–
Drinking, *n* (%)	6 (28.57)	2 (33.33)	0.822	–	–
FBDS, *n* (%)	8 (38.10)	3 (50.00)	0.602	–	–
Cognitive impairment, *n* (%)	10 (83.33) (9 missed)	4 (80.00) (1 missed)	0.870	–	–
Sleep disorders, *n* (%)	9 (42.86)	4 (66.67)	0.313	–	–
CSF oligoclonal band, *n* (%)	11 (52.38)	3 (50.00)	0.918	–	–
CSF WBC count, cells/uL	7.00 (4.50–20.50)	4.50 (3.50–150.00)	0.305	–	–
CSF protein, mg/dL	43.31 (29.40–53.17)	48.35 (35.94–139.60)	0.068	0.169	1.033 (1.003–1.083)
CSF antibody titer	32 (10–32)	10 (5–21)	0.162	0.104	0.920 (0.784–0.986)
Serum antibody titer	66 (10–100)	32 (21–66)	0.403	–	–
Abnormal EEG, *n* (%)	16 (76.19)	5 (83.33)	0.773	–	–
SUVR (ACC)	1.26 (1.14–1.37)	1.03 (1.01–1.15)	0.032	0.043	2.139e−13 (4.538e−30–2.172e−7)
SUVR (MCC)	1.42 (1.23–1.52)	1.29 (1.22–1.35)	0.227	–	–
SUVR (PCC)	1.21 (1.08–1.34)	1.12 (0.93–1.23)	0.272	–	–
Tumor, *n* (%)	1 (4.76)	2 (33.33)	0.628	–	–
mRS (follow-up)	0 (0–2) (4 missed)	1 (0–2) (1 missed)	0.782	–	–

Categorical variables were recorded as *n* (%). Continuous variables were recorded as median (IQR)

*ACC* anterior cingulate cortex, *CSF* cerebrospinal fluid, *EEG* electroencephalography, *FBDS* faciobrachial dystonic seizure, *MCC* midcingulate cortex, *mRS* modified Rankin scale, *PCC* posterior cingulate cortex, *WBC* white blood cell

## Discussion

In this study, we reported a cohort of 49 patients with antibody-confirmed AE and investigated the role of the cingulate cortex and potential risk factors for poor psychiatric prognosis in AE-PS. Compared with AE-WPS patients, AE-PS patients showed decreased metabolism and perfusion in the cingulate cortex, suggesting that cingulate cortex metabolic/perfusion dysfunction may be associated with the pathology of psychiatric symptoms in AE. Furthermore, we found that the metabolic dysfunction of ACC was independently correlated with a higher risk of poor psychiatric outcomes.

Alteration in mental function is one of the cardinal symptoms of AE. Psychiatric symptoms were reported in more than half of the AE patients in this study, which is consistent with previous reports [19]. The corresponding psychiatric symptoms could be hallucinations, delusions, agitation, thought blocking, catatonia, and a depressed mood. Among these, agitation and thought blocking are the most common psychiatric symptoms, except for anti-GAD65 encephalitis. This study uncovered a nonspecific presentation of psychiatric features in the adult AE population. Moreover, different subtypes may have different psychiatric presentations. The most common AE with psychiatric disorders is anti-GABAB encephalitis. However, small subtype-specific sample sizes prevented us from exploring specific psychiatric symptoms in different AE subtypes. In a future study, we plan to add more statistical analysis with a larger sample size to make our results more conclusive.

We found significant differences between the two AE groups regarding smoking history and sleep disorders. The mechanism and the association of smoking with AE-PS remain largely unclear. Tobacco can lead to a disturbance in the levels of certain neurotransmitters [20], and may also influence the activity of T and B lymphocyte subpopulations and promote cytokine-driven systemic inflammation, further contributing to autoimmune diseases [21]. An abnormal immune response and neurotransmitter imbalance can attack psychiatric-related neurons or structures that may lead to AE-related psychiatric symptoms. Psychiatric disorders and sleep disturbances are prominently related. An early sign of psychiatric disorder is often a sleep disturbance, which can then contribute to the presentation of psychiatric disorders [22]. Although frequent psychiatric symptoms might be influenced by impaired brain regions involved in the psychiatric network in AE patients, the exact underlying neuroanatomical and pathological mechanism behind AE-PS is still unknown. Our findings from the aspect of brain metabolism and perfusion using ^18^F-FDG-PET and ASL indicate that, compared with AE-WPS patients, AE-PS patients exhibited high hypometabolism and hypoperfusion in the cingulate cortex. This result suggests that the cingulate cortex may be a key region in AE-PS. Notably, cingulate cortex hypometabolism and atrophy have been concurrently reported in AE cases, indicating that cortex atrophy may be a potential mechanism of hypometabolism, especially after chronic neuroinflammation [23, 24].

Some patients still suffered a poor psychiatric prognosis during long-term follow-up, imposing a burden on the patient’s family and society. Our results showed that the poor psychiatric prognosis of AE was positively related to SUVR of the ACC, which is associated with complex cognitive functions such as empathy, impulse control, emotion, and decision-making [25]. Impaired ACC function has been linked to psychopathology and emotional dysregulation [26]. Timely monitoring of PET–CT may provide insights into this complicated disease state and help improve the psychiatric prognosis.

Anti-psychotic therapies may still be used due to the prominent psychiatric symptoms. In our cohort, three of the patients received psychotropic medication without beneficial effects. Fortunately, these three patients later received immunotherapies, and all had good psychiatric outcomes. Previous studies showed that resistance to anti-psychotic medication is a feature of the psychiatric symptoms of anti-NMDAR encephalitis [27]. We, therefore, believe that immunotherapy can be a more efficient method of treating psychiatric symptoms in AE. A further large-scale prospective study is required to confirm these inferences.

Nonetheless, this study had a few limitations. First, the identification of psychotic symptoms has mainly relied on the clinician’s assessment, which can be subjective in nature. The absence of a quantifiable scale for symptom severity limits the correlation analysis between clinical severity and radiographic severity. Second, we could not fully analyze the different effects between anti-psychotic therapies and immunotherapies. We could not completely avoid the impact of therapy on PET scans because of the data limitation. Third, current PET and ASL techniques offer a limited characterization of the perfusion and metabolic brain properties. A further large-scale prospective study would be required to confirm these findings.

## Conclusion

In summary, psychiatric symptoms occur in more than half of AE patients. Impaired cingulate cortex function may be the potential cause of psychiatric symptoms in AE. Hypometabolism of ACC is an independent prognostic factor predicting an unfavorable psychiatric prognosis.

### Supplementary Information

Below is the link to the electronic supplementary material.

Supplementary file1 (TIFF 4848 KB)

## Data Availability

All data are available upon reasonable request.
