# Supplementary figures and images for: Cerebral glucose hypometabolism and hypoperfusion of cingulate gyrus: an imaging biomarker of autoimmune encephalitis with psychiatric symptoms
Source: J Neurol. 2023 Nov 9;271(3):1247–55. doi: 10.1007/s00415-023-12051-z (PMC10896782; doi:10.1007/s00415-023-12051-z)

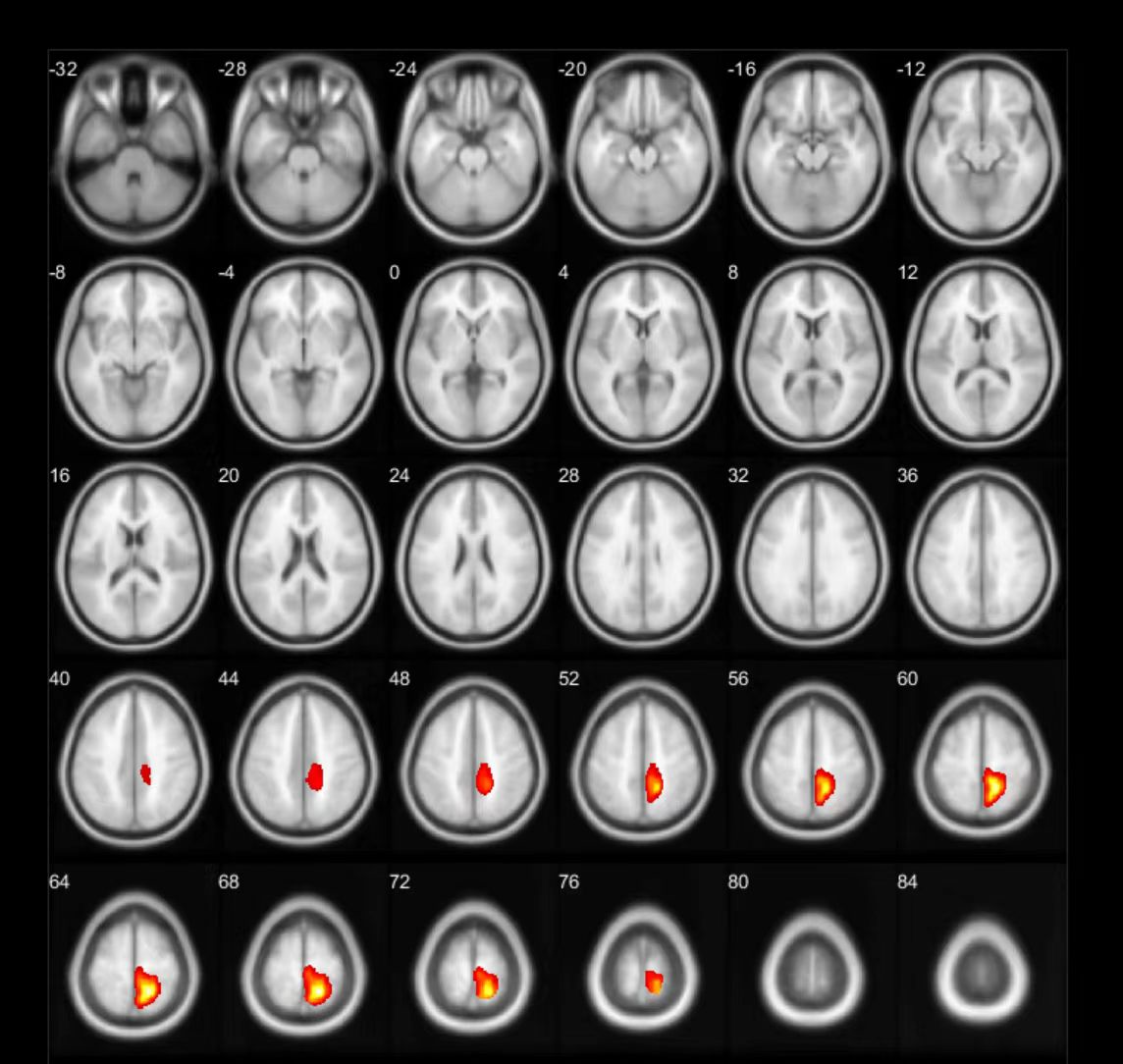

Supplement: Supplementary file 1 — Supplementary file1 (TIFF 4848 KB) [file 415_2023_12051_MOESM1_ESM.tiff]
